# Combinations of Tibetan tea and medicine food homology herbs: A new strategy for obesity prevention

**DOI:** 10.1002/fsn3.3081

**Published:** 2022-09-28

**Authors:** Ye Yuan, Bin Zhang, Jing‐liu He, Ting Wei, De‐jun Liu, Wen‐jun Yang, Cheng‐Yi Guo, Xu‐qiang Nie

**Affiliations:** ^1^ Department of Pharmacy & Medical Laboratory Ya'an Polytechnic College Ya'an China; ^2^ College of Pharmacy Zunyi Medical University Zunyi China; ^3^ Key Laboratory of the Basic Pharmacology of the Ministry of Education Zunyi Medical University Zunyi China; ^4^ Joint International Research Laboratory of Ethnomedicine of Chinese Ministry of Education Zunyi Medical University Zunyi China

**Keywords:** hypercaloric high‐fat diet (HFD), intestinal flora, obesity, safety evaluation, Winter Melon Lotus Leaf Tibetan Tea (WLTT)

## Abstract

Obesity has become a significant global public health problem. Functional drinks have been an essential direction for obesity prevention research. The present study investigated the preventive effect and safety of winter melon and lotus leaf Tibetan tea (WLTT, a compound tea drink based on Ya'an Tibetan Tea and medicine food homology herbs) on obesity. The rats' hypercaloric high‐fat diet (HFD) obesity model was established to evaluate obesity prevention and explored the mechanism through intestinal flora regulation. The results showed that in obese rats with the intervention of WLTT (400, 800, and 1600 mg/kg BW), the body weight, fat accumulation, adipocyte cell size, serum lipid levels, and antioxidant enzyme activity (SOD, GSH‐Px, and MDA) were progressively improved. 16S rRNA high‐throughput sequencing showed that WLTT could improve intestinal flora disorders due to HFD, which significantly reversed the relative abundance of *Firmicutes* and the *F/B* ratio associated with an HFD, and significantly upregulated the relative abundance of *Verrucomicrobia*. At the genus level, the downregulation of the relative abundance of *Akkermansia* and *unclassified_Lachnospiraceae* groups, and the upregulation of the relative abundance of *Romboutsia, Ruminococcus, Corynebacteriume*, and *Saccharibacteria_genera_incertae_sedis* groups brought about by the HFD were significantly reversed. The results of the above experiments were compared favorably with those of a parallel experiment with Bi ‐Sheng ‐Yuan slimming tea (BSY, a functional drink based on green tea and medicine food homology herbs). Overall, the findings have provided that WLTT can prevent obesity owing to an HFD by regulating intestinal flora and has a good safety profile, and combinations of Tibetan tea and medicine food homology herbs could be a new option for obesity prevention.

## Graphical abstract

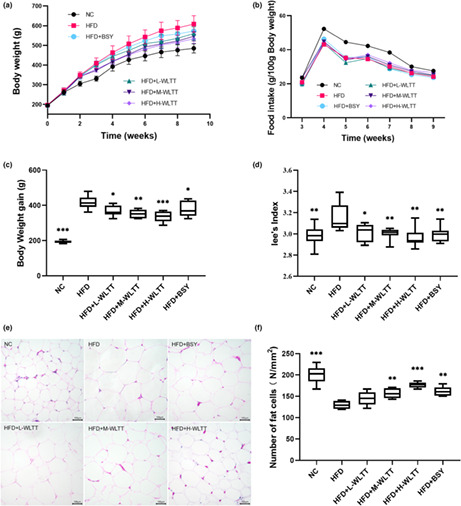

## 1 INTRODUCTION

With economic development, rich diets, and changes in lifestyle habits, the prevalence of obesity increases annually. According to the latest national prevalence estimates for 2015–2019, 6.8% were overweight and 3.6% obese in children under 6 years old, 11.1% overweight, and 7.9% obese in children and adolescents aged 6–17 years old, 34.3% overweight and 16.4% obese in adults (≥18 years), and with a population base of 1.41 billion, the obesity situation in China is no longer optimistic (NCD Risk Factor Collaboration [NCD‐RisC], 2016; Pan et al., 2021). Many studies have shown that obesity is a high‐risk factor for many chronic diseases such as diabetes, hypertension, hyperlipidemia, and coronary heart disease, and is also a paramount factor in increasing cerebrovascular disease, increasing the load on the heart, causing bone and joint disease, and predisposing to cancer (Banerjee et al., 2020; Marini et al., 2020; Oliveira et al., 2020; Seravalle & Grassi, 2017). Improving obesity and losing weight has become a long‐standing wish of obese patients worldwide. However, because there is no specific medicine for obesity, the lack of long‐term self‐discipline required for diet control and physical exercise is why most obese patients fail to lose weight (Carbone et al., 2019). Thus, the search for and development of food‐grade safe and healthy beverages for long‐term prevention may be an essential step toward improving the situation described above.

Tea is known as one of the safest and healthiest beverages globally and is consumed by people worldwide (Chen et al., 2015; Chung et al., 2020; Li et al., 2019; Liu et al., 2022; Shen et al., 2019; Vieux et al., 2019). Ya'an Tibetan Tea is a classic tea variety fermented in the Ya'an region of Sichuan Province in China with a unique geographical environment with exquisite traditional techniques and has a history of over 1300 years (Chen, 2017). Numerous studies have confirmed that Ya'an Tibetan Tea has a wide range of biological activities in terms of antioxidants, regulation of obesity, and related metabolic syndrome. (Liu et al., 2022; Wang, Wu, et al., 2021; Xie et al., 2018; Zheng et al., 2020; Yuan et al., 2016). In order to enrich the flavor and taste of Ya'an Tibetan Tea and enhance its functionality, the research team has developed WLTT based on Ya'an Tibetan Tea, using the traditional Chinese medicine compounding concept and incorporating medicine food homology herbs. In order to explore the modulating effect of WLTT on obesity and its potential mechanism, the group studied the intervention effect of WLTT on obesity in a high‐calorie model of obese rats and evaluated its safety, and analyzed its potential mechanism through the regulation of intestinal flora disorder. At the same time, the team conducted a comprehensive and systematic comparative study of BSY, which is mainly green tea and herbal medicine with a large customer base in China, to fully explore the feasibility of combining Tibetan tea and medicine food homology herbs.

## 2 MATERIALS AND METHODS

### 2.1 Materials

Winter Melon and Lotus Leaf Tibetan Tea (WLTT), formulated with Lotus Leaf, Winter Melon, Cassia Seed, Hawthorn, Chen Pi, Mulberry, Puzzle Nut, and Ginseng, was offered by Sichuan Ya'an Yixing Tibetan Tea Co., Ltd. Bi‐Sheng‐Yen slimming tea (BSY), composed of senna, green tea, cassia seed, lotus leaf, and alisma, was purchased from Beijing Autoshure Health Products Development Co., Ltd. (Lot No. 19201102). Superoxide dismutase (SOD), malondialdehyde (MDA), and glutathione peroxidase (GSH‐Px) kits were purchased from Nanjing Jiancheng Institute of Biological Engineering. Chloral hydrate and formaldehyde were all analytically pure and purchased from Sinopharm Chemical Reagent Co., Ltd.

### 2.2 Preparation of WLTT and BSY

Winter Melon and Lotus Leaf Tibetan Tea was converted to a medium dose of 800 mg/kg in experimental rats based on the recommended daily human dose of 8 g and then designed for a low dose of 400 mg/kg and a high dose of 1600 mg/kg. WLTT was decocted for 20 min at a tea‐to‐water ratio of 1:40 for the first decoction and for 10 min at a tea‐to‐water ratio of 1:20 for the second decoction to obtain the tea broth, which was concentrated to the corresponding raw tea concentration for both decoctions based on a gavage volume of 10 ml/kg for rats. BSY was converted to an experimental dose of 500 mg/kg in rats based on the recommended daily human dose of 5 g. The decoction method is the same as for WLTT and is also concentrated to the corresponding raw tea concentration based on the rats' gavage volume. The above samples were decocted once every day, concentrated, and then refrigerated at 4°C.

### 2.3 Animal experimental protocol

Seventy SPF grade SD male rats, weighing 200 ± 20 g, were purchased from Chengdu Dashuo Experimental Animal Co. Ltd. (license number: SCXK [Chuan] 2015–030). High‐calorie feed formula: 50% basal feed, 15.0% sucrose, 15.0% lard, 10% casein, 5% whole milk powder, 2% calcium hydrogen phosphate, 1.9% microcrystalline cellulose, 1.1% rock flour. Calories are 4376.4 kcal/kg, of which 19.52% are protein calories, 40.16% are fat calories, and 40.32% are carbohydrate calories‐formulated and supplied by Jiangsu Synergy Pharmaceutical Bioengineering Co., Ltd. (production license: Su Feeding Certificate 2014–01008).

According to the method for functional evaluation of healthy food, 70 SPF‐rated SD male rats were taken and fed for 1 week after acclimatization. The rats were randomly divided into two groups according to body weight, 10 of which were normal control (NC) and given maintenance chow, and the remaining 60 were a hypercaloric high‐fat diet model group and given hypercaloric chow. After 2 weeks of feeding, 60 rats given hypercaloric chow were sorted by weight gain, and after eliminating the obese‐resistant rats with lower weight gain, the screened 40 obese‐sensitive rats were again randomly allocated into five experimental groups with 8 rats each according to their body weight range. They were hypercaloric high‐fat diet model group (HFD), Bi‐Sheng‐Yuan slimming tea 500 mg/kg (HFD + BSY), WLTT 400 mg/kg group (HFD + L‐WLTT), WLTT 800 mg/kg group (HFD + M‐WLTT), and WLTT 1600 mg/kg group (HFD + H‐WLTT). Each experimental group was given the corresponding concentration of the test sample, and the NC group and the model group were given double‐distilled water as the control. All rats were oral gavage at a dose of 10 ml/kg once a day, and the dose was adjusted by weighing once a week. All animal experiments were conducted with the Ethics Committee of Zunyi Medical University (approval no: ZMUER2020‐1‐057).

### 2.4 Sample collection and biochemical index and histological analysis

At the end of the intervention cycle, the rats were fasted without water for 12 h, weighed, anesthetized with 10% chloral hydrate by intraperitoneal injection, and collected blood from the femoral artery. After the plasma was left to stand for 2 h, the serum was separated by centrifugation at 3000 r/min at 4°C for 10 min, and taking a partial serum sample was quickly sent to the Laboratory Department of Ya'an Hospital of Traditional Chinese Medicine in Sichuan Province for biochemical indexes such as TG, TC, HDL‐C, LDL‐C, AST, ALT, BUN, and Cre. According to the kit instructions, another part of the serum sample will be analyzed for the antioxidant enzymes SOD, MDA, and GSH‐Px (Nanjing Jiancheng Institute of Biological Engineering). After blood collection, the rat's body length was measured, and Lee's index was calculated according to the formula, Lee's index = $\sqrt[{3}]{\text{body weight}\left(g\right)}\times 10÷$ body length (cm). The rat was quickly dissected, and the contents were quickly removed from the same part of the cecum in sterile lyophilization tubes and placed in an insulated box with dry ice for freezing. The liver, kidney, perirenal fat, and epididymal fat tissue were removed and weighed on an electronic balance to calculate the organ index. After weighing, appropriate amounts of liver and epididymal adipose tissue were cut and fixed in formalin fixative and sent to the Department of Pathology, Affiliated Hospital of Sichuan Ya'an Vocational College for pathological analysis. The rat adipose tissue change was observed by H&E staining. Six adipose tissue sections were randomly selected from each group and photographed at 200× field of view to count the number of fat cells in the same area.

### 2.5 16S rRNA high‐throughput sequencing of gut microbiota of cecum

The cecum contents of three rats were randomly collected from each group and sent to Sangon Biotech (Shanghai) Co., Ltd. for high‐throughput sequencing library construction and Illumina Mi Seq sequencing. The raw data obtained based on sequencing were stitched according to overlap relationships. The samples were differentiated and then quality‐controlled and filtered for sequence quality, followed by OTU clustering and species annotation, Alpha diversity analysis, Beta diversity analysis, grouping test analysis, and species taxonomic composition analysis.

### 2.6 Statistical analysis

The experimental data were statistically analyzed using GraphPad Prism 8.0 software (GraphPad Prism Software). One‐way analysis of variance was used for comparison between groups, and data are expressed as $\bar{X}$ ± *SD*. Differences of *p* < .05 were considered statistically significant.

## 3 RESULTS

### 3.1 Effect of WLTT and BSY on body weight, food intake, Lee's index and fat cells

**FIGURE 1:**
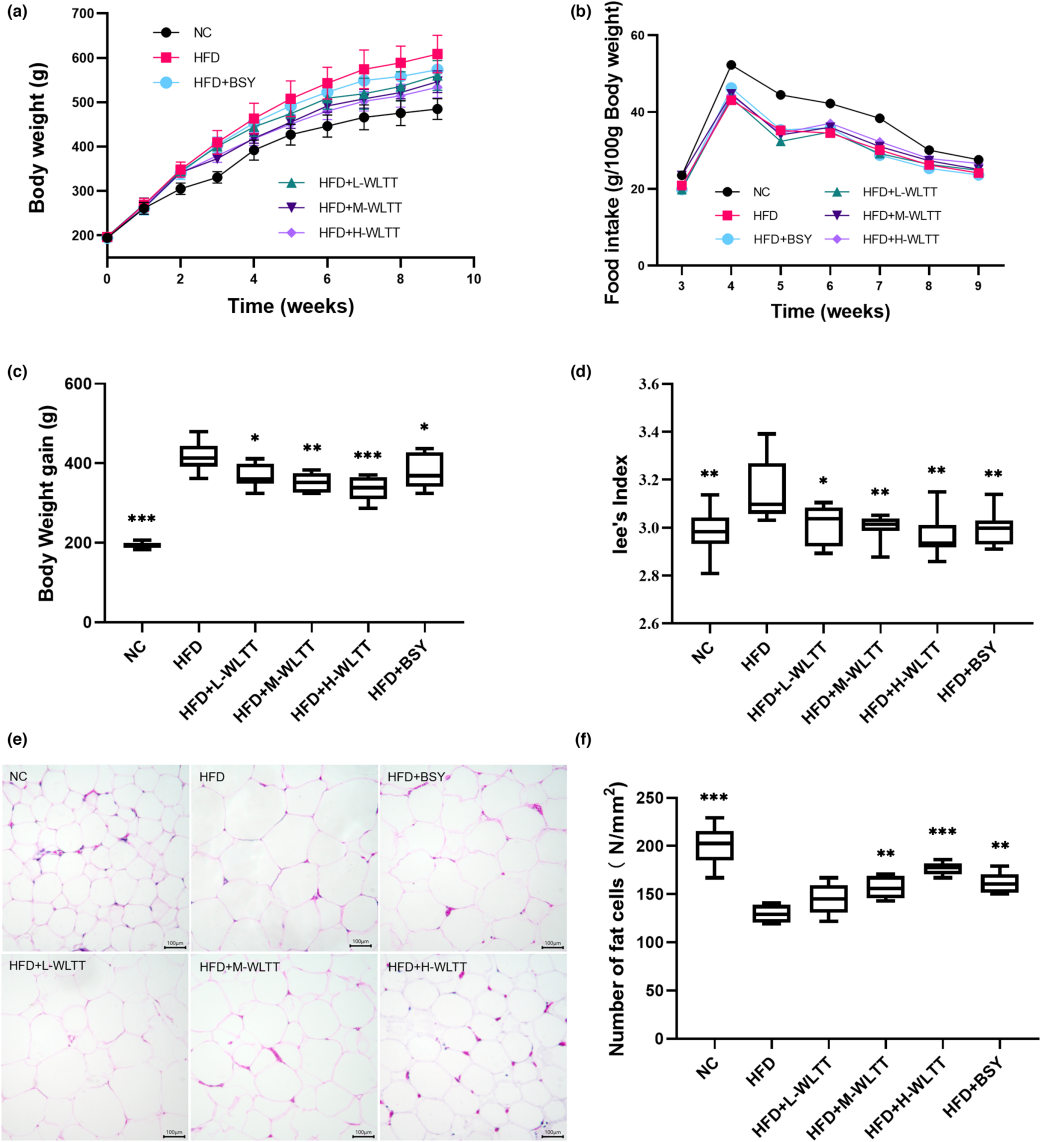
The effects of body weight, food intake, Lee's Index and fatty tissue H&E staining in obese rats. (a) body weight, (b) food intake, (c) body weight gain, (d) Lee's Index, (e) epididymal fatty tissue H&E staining (×200), (f) number of fat cells per square millimeter in 200× field. NC, normal control group; HFD, hypercaloric high‐fat diet group; HFD + L‐WLTT, HFD + Winter Melon Lotus Leaf Tibetan Tea 400 mg/kg group; HFD + M‐WLTT, HFD + Winter Melon Lotus Leaf Tibetan Tea 800 mg/kg group; HFD + H‐WLTT, HFD + Winter Melon Lotus Leaf Tibetan Tea 1600 mg/kg group; HFD + BSY, HFD + Bi‐Sheng‐Yuan slimming tea 500 mg/kg group. Data were expressed as a mean ± *SD*, *n* = 8, compared with HFD group, *p < .05, **p < .01, ***p < .001

After 2 weeks on a hypercaloric diet, rats showed significant weight differentiation, thus demonstrating the model's success. However, with the intervention of the experimental samples, the weight of each experimental rat further diverged. The weight‐loss trend was significantly better in each experimental group of WLTT than in the BSY group, showing a dose‐dependent trend (*p* < .05, Figure 1a). However, the results of monitoring the change in food intake of each experimental group weekly showed that after the experimental groups were fed high‐fat chow, there was a tendency for the food intake of each group to decrease. Luckily, there was no difference between each sample intervention group and the HFD group, and the food intake curve fit (Figure 1b). In addition, statistical analysis of the weight gain in each group for the experimental cycle showed similar results (*p* < .05, Figure 1c). Analysis of Lee's index in rats revealed that both the WLTT and BSY groups down‐regulated Lee's index in obese rats (*p* < .05, Figure 1d). Morphological analysis of H&E staining of rat fatty tissue showed significant variability in the number and size of adipocytes after H&E staining and photographic analysis at the same field‐of‐view magnification. NC and HFD groups showed the most significant cellular variability, while the rest of the experimental groups showed dose‐dependent variability, and morphological analysis of the HFD + M‐WLTT and HFD + H‐WLTT groups had some advantage over the BSY group (*p* < .05, Figure 1e,f).

### 3.2 Effect of WLTT and BSY on visceral fat, blood lipids, and antioxidant enzymes

Analysis of body fat content showed a significant decrease in epididymal fat weight, perirenal fat weight, and fat coefficient in all experimental groups of WLTT (*p* < .05, Table 1). In contrast, there was no statistical difference between the BSY group in terms of perirenal fat weight and fat coefficient (*p* > .05, Table 1). The effect of WLTT and BSY on lipids was found to be mainly in TG, which was statistically different in all experimental groups (*p* < .05, Table 1), with statistics showing that the HFD + L‐WLTT and HFD + M‐WLTT groups were superior to the BSY group. As for the rest of the lipid parameters, none of the WLTT experimental groups showed statistical differences from the BSY group (*p* > .05, Table 1), but the data showed a slight downward trend in TC and LDL‐C upward trend H‐DLC in each experimental group. The antioxidant enzyme assay results showed that the HFD + M‐WLTT and HFD + H‐WLTT groups could upregulate serum SOD and GSH‐Px and downregulate serum MDA in the high‐calorie‐model rats, with statistically significant differences (*p* < .05, Table 1). In contrast, the BSY group in the parallel experiment showed no significant difference in the effects on GSH‐Px and MDA, except for a slight increase in serum SOD (*p* > .05, Table 1).

**TABLE 1:** The effect of blood lipids, visceral fat and antioxidant enzymes in obese rats

Parameters	NC	HFD	HFD + L‐WLTT	HFD + M‐WLTT	HFD + H‐WLTT	HFD + BSY
Epididymal fat weight (g)	9.48 ± 1.83***	18.28 ± 3.40	14.27 ± 3.02**	12.66 ± 1.79***	11.43 ± 1.20***	14.96 ± 2.42*
Perirenal fat weight (g)	9.11 ± 1.76***	19.83 ± 5.31	14.81 ± 3.01*	14.18 ± 3.78**	10.43 ± 2.41***	17.09 ± 3.63
Fat coefficient (100%)	4.00 ± 0.65***	6.45 ± 0.98	5.41 ± 0.93*	5.16 ± 0.87**	4.28 ± 0.71***	5.86 ± 0.96
TG (mmol/ml)	0.66 ± 0.22**	1.13 ± 0.41	0.66 ± 0.22**	0.45 ± 0.17***	0.76 ± 0.16*	0.71 ± 0.31*
TC (mmol/ml)	1.43 ± 0.21	1.61 ± 0.22	1.53 ± 0.28	1.54 ± 0.23	1.50 ± 0.34	1.44 ± 0.32
LDL‐C (mmol/ml)	0.36 ± 0.07	0.41 ± 0.12	0.40 ± 0.03	0.37 ± 0.09	0.36 ± 0.10	0.34 ± 0.08
HDL‐C (mmol/ml)	0.92 ± 0.20	0.75 ± 0.14	0.70 ± 0.27	0.83 ± 0.18	0.97 ± 0.16	0.75 ± 0.19
SOD (U/ml)	208.65 ± 21.65**	167.42 ± 17.37	185.39 ± 20.31	199.49 ± 32.81*	213.63 ± 18.12**	191.27 ± 26.95*
MDA (nmol/ml)	4.76 ± 1.05**	8.85 ± 0.69	6.38 ± 2.89	5.19 ± 1.48*	4.38 ± 1.18**	6.96 ± 3.03
GSH‐Px (U/ml)	2407.84 ± 555.79**	1449.02 ± 520.86	1809.80 ± 511.92	2017.65 ± 350.01*	2043.14 ± 252.51*	1517.65 ± 380.78

*Note*: Data were expressed as a mean ± *SD*, *n* = 8, compared with HFD group.

Abbreviations: GSH‐Px, glutathione peroxidase; HDL‐C, high‐density lipoprotein cholesterol; HFD + BSY, HFD + Bi‐Sheng‐Yuan slimming tea 500 mg/kg group; HFD + H‐WLTT, HFD + Winter Melon Lotus Leaf Tibetan Tea 1600 mg/kg group; HFD + L‐WLTT, HFD + Winter Melon Lotus Leaf Tibetan Tea 400 mg/kg group; HFD + M‐WLTT, HFD + Winter Melon Lotus Leaf Tibetan Tea 800 mg/kg group; HFD, hypercaloric high‐fat diet group; LDL‐C, low‐density lipoprotein‐cholesterol; MDA, malondialdehyde; NC, normal control group; SOD, superoxide dismutase; TC, total cholesterol; TG, triglycerides.

* *p* < .05; **p < .01; ***p < .001.

### 3.3 Effect of WLTT on liver and kidney function

**FIGURE 2:**
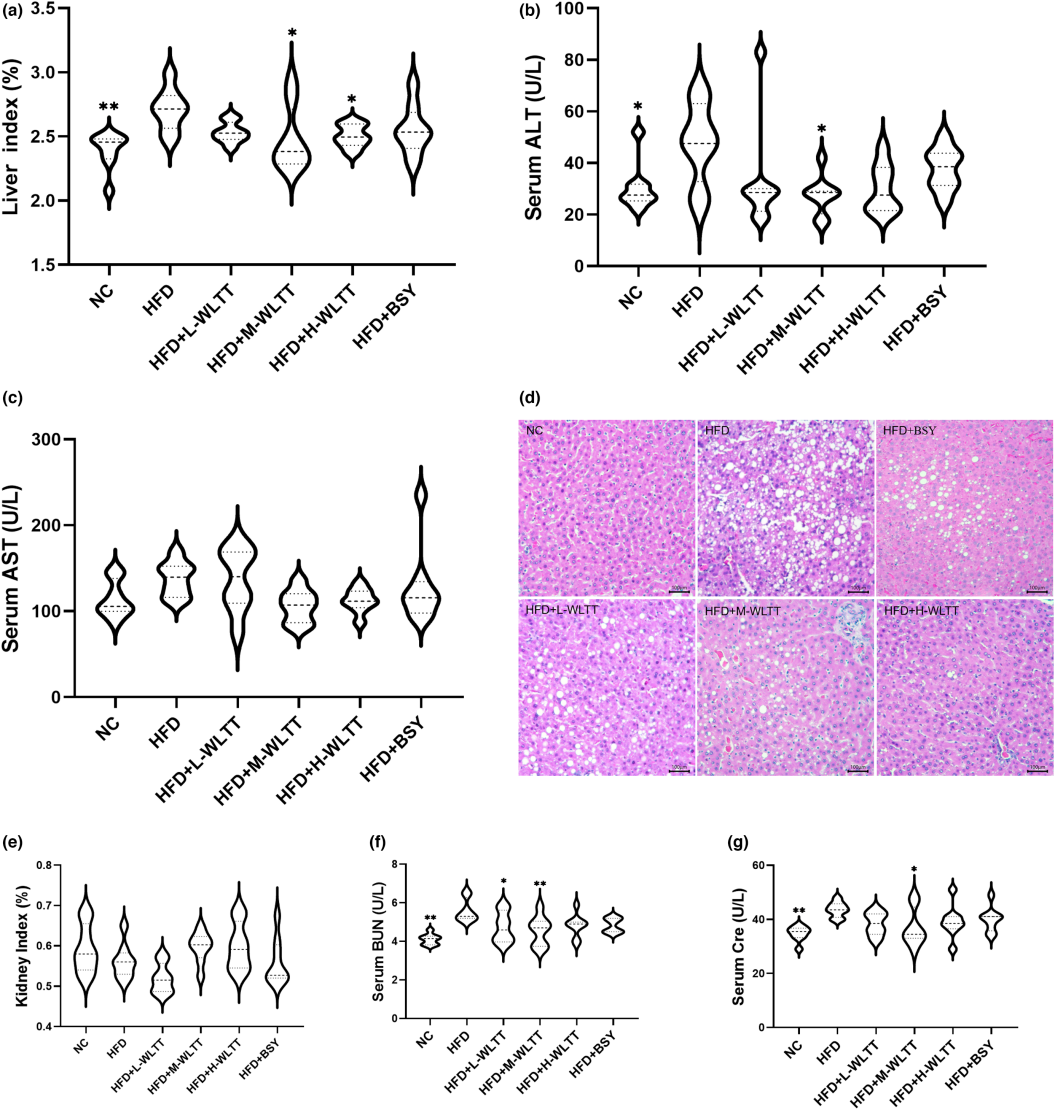
The effect of liver and kidney function in obese rats. (a) Liver index; (b) ALT, alanine aminotransferase; (c) AST, aspartate aminotransferase; (d) Liver tissue H&E staining (×200); (e) Kidney index; (f) BUN, blood urea nitrogen; (g) Cre, creatinine. Data were expressed as a mean ± *SD*, *n* = 8, compared with HFD group, *p < .05, **p < .01

The safety of WLTT was also of great interest to the group. The results showed that WLTT significantly downregulated the liver index and ALT in obese rats (*p* < .05, Figure 2a,b), while the AST was downregulated but not statistically different (*p* > .05, Figure 2c). H&E staining analysis of liver tissue showed that the fat vacuole area was significantly lower in the WLTT experimental groups than in the HFD group in a dose‐dependent manner (Figure 2d). Meanwhile, the BSY group showed no statistical difference in the liver index, ALT, and AST compared with the model group (*p* > .05, Figure 2a–c), and the area of fat vacuoles in the liver tissue was slightly reduced (Figure 2c). In terms of renal function, there was no statistical difference between the WLTT dose group and the BSY group in terms of the renal index compared to the HFD group (*p* > .05, Figure 2e–g), but the HFD + M‐WLTT group had a downregulatory effect on BUN and Cre (*p* < .05, Figure 2f,g), whereas the BSY group showed no statistical difference in this regard.

### 3.4 Effect of WLTT and BSY on the intestinal flora

**FIGURE 3:**
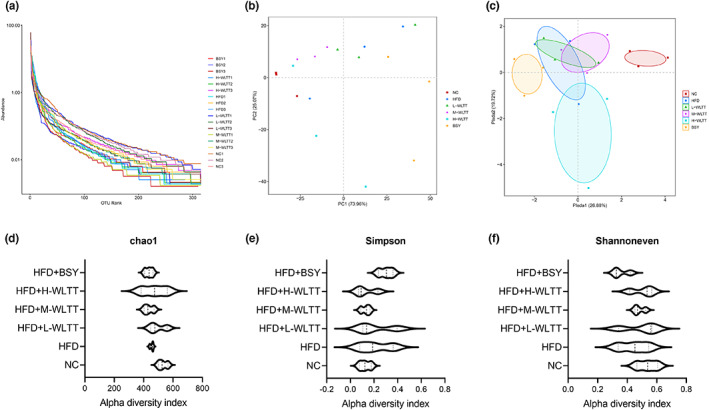
The diversity analyses of intestinal flora in obese rats. (a) Rank‐abundance graph; (b) Species principal component analysis plots; (c) Partial least squares discriminant analysis plots; (d) chao1 index analysis graph, (e) Simpson Index analysis graph (f) Shannoneven Index analysis graph.

Analysis of the 16S rRNA gene sequencing showed that the sequencing depth was primarily sufficient to cover the samples' strain information. The Rank‐abundance curve is wide and flat, indicating a good diversity of intestinal microflora (Figure 3a). Beta diversity analysis of species principal component analysis analyzes the more similar the species composition of the sample (Figure 3b). The subgroup tests' partial least squares discriminant analysis showed differences in microbial communities between groups (Figure 3c). Compared to the NC group, the PLS‐DA analysis of each microbiota group was biased by the intervention of the hypercaloric model with WLTT and BSY, with the WLTT groups being closer together than the NC group. For diversity index analysis, there was an increase in Chao1 and Shannon indexes and a decrease in Simpson index for the gut microbiota with WLTT intervention compared to the HFD group, but none of them were statistically different (*p* > .05, Figure 3d–f).

**FIGURE 4:**
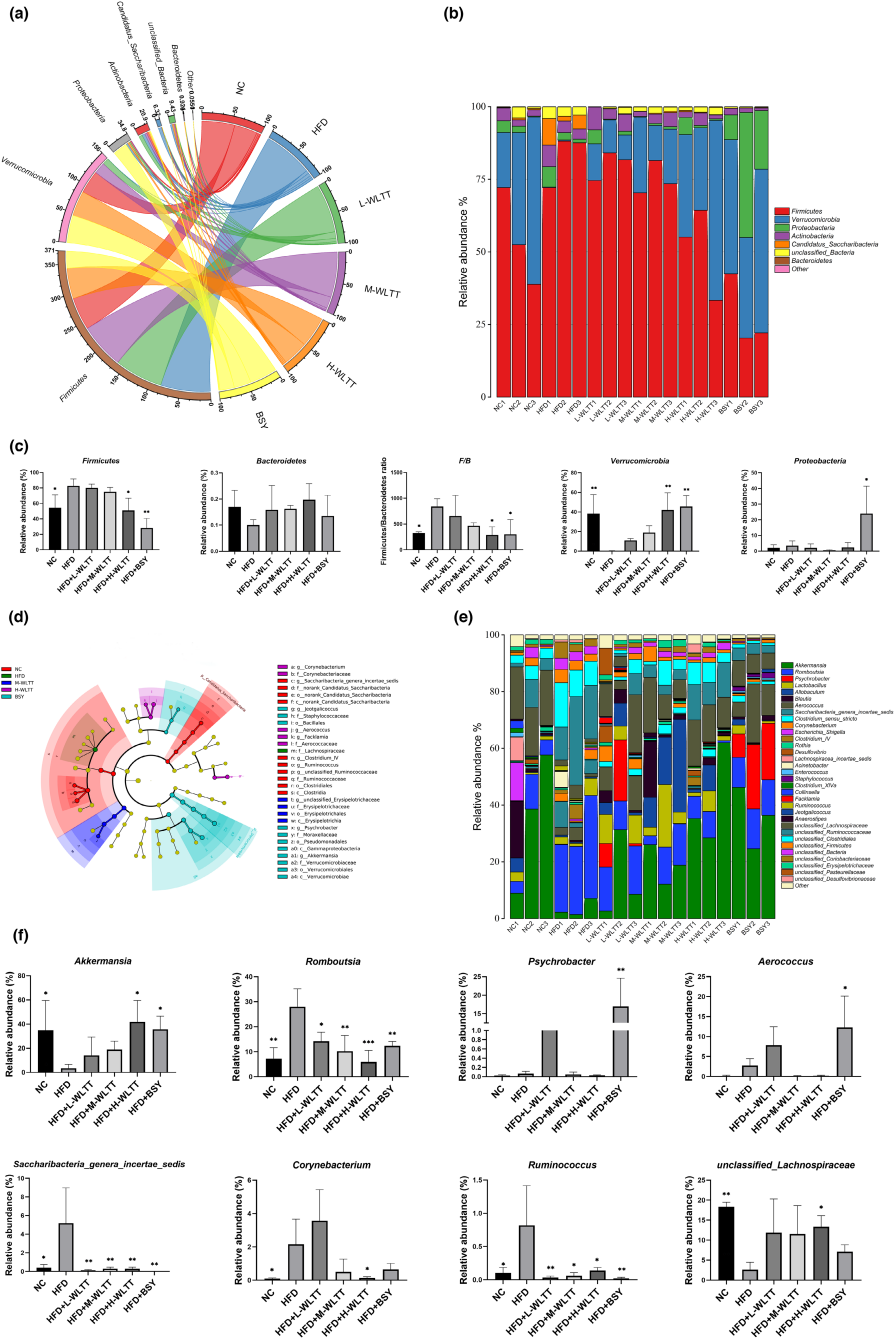
The modulatory effects of intestinal flora in obese rats. (a) Circos plots at the phylum level; (b) Relative species abundance between groups plots at the phylum level; (c) Intergroup phylum level species variability analysis plots at the phylum level; (d) Ring tree diagram for discriminant analysis of differences between LEfSe (Linear discriminant analysis Effect Size) groups at the genus level; (e) Relative species abundance between groups plots at the genus level; (f) Intergroup genus level species variability analysis plots.

In the analysis of the taxonomic composition of microbial species, the Circos plot on phylum level (Figure 4a) and the relative abundance of species between groups on phylum level (Figure 4b) responded in different dimensions to the changes in the abundance of the dominant microbial population composition of each experimental group under the intervention of the high‐heat model and different experimental samples. The results showed that the WLTT experimental groups tended to significantly downregulate the *Firmicutes* and the *F/B* ratio at the phylum level while simultaneously upregulating *Verrucomicrobia*, with HFD + H‐WLTT performing relatively well and showing statistical differences (*p* < .05, Figure 4c). This result also clearly suggests that WLTT at the phylum level has the potential to reverse the intestinal flora disorders associated with the hypercaloric model. Meanwhile, BSY also showed some ability to improve intestinal flora disorders, but the results in the Proteobacteria were not as good, with a statistically significant upregulation compared to the model group (*p* < .05, Figure 4c).

In further analysis of the taxonomic composition of microbial species, the circular dendrogram for discriminant analysis of differences between LEfSe (Linear discriminant analysis Effect Size, http://huttenhower.sph.harvard.edu/lefse/) groups provides a more visual representation of the differences in microbial communities between groups (Figure 4d). The relative abundance of species between groups (Figure 4e) further reflects changes in the abundance of the dominant microbial groups in each experimental group at the genus level composition under the intervention of the high‐heat model with WLTT and BSY in a multidimensional manner. The results showed that the HFD + H‐WTLL group upregulated the relative abundance of the genus *Akkermansia*, *unclassified_Lachnospiraceae* (*p* < .05, Figure 4f) compared to the HFD group, while significantly downregulating the *Romboutsia* (*p* < .01, Figure 4f), *Saccharibacteria_genera_incertae_sedis* (*p* < .05, Figure 4f), *Corynebacterium* (*p* < .05, Figure 4f) and *Ruminococcus* (*p* < .05, Figure 4f). The BSY group also showed some intervention in the above flora. However, the overall evaluation was not as good as that of the HFD + H‐WTLL group, especially in the relative abundance of *Psychrobacter* (*p* < .01, Figure 4f) and *Aerococcus* (*p* < .05, Figure 4f) showed a significant upregulation trend. However, the WTLL experimental groups did not show statistical differences (Figure 4f).

**FIGURE 5:**
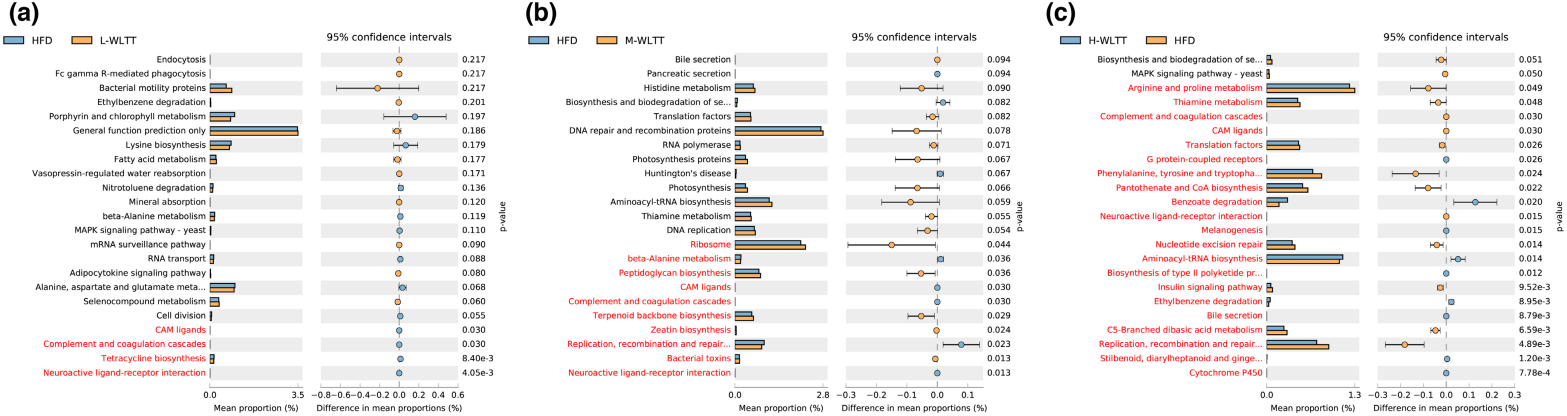
The effect of the function of microbial communities predicted by PICRUST. The left‐hand side of each graph shows the proportion of abundance of different functional categories in the two samples, the middle shows the proportion of differences in functional abundance within the 95% confidence interval, and the far right‐hand side shows the p‐value, with *p* < .05 indicating a significant difference, and is marked in red.

In order to explore the effect of the WLTT on the regulation of intestinal flora and thus potential functions, the team compared the species composition obtained from sequencing with the KEGG PATHWAY database to infer the composition of functional genes in the samples. STAMP differences between the WLTT intervention groups and the HFD group were analyzed by Welch's *t*‐test and found that the WLTT interventions resulted in altered functional microbial abundance compared to the HFD group and that the number of statistically different functional metabolic pathways increased progressively with increasing dose. It mainly causes changes in some metabolic pathways, such as amino acid, insulin, and bile metabolism, altering the biosynthesis and degradation of some secondary metabolites (Figure 5).

## 4 DISCUSSION

The worsening trend of global obesity cases has been regarded as the epidemic of the 21st century, and medical and nutritional experts around the world are working hard to address this problem. The development of functional drinks from natural foods that intervene in obesity has been an important direction in obesity prevention (Rodríguez‐Pérez et al., 2019; Shang et al., 2021; Wharton et al., 2020; Xu et al., 2021). However, achieving the desired effect of preventing and treating obesity in traditional tea drinks may be challenging. The fusion of a relatively inclusive tea with traditional medicine food homology herbs, and the concept of compounding, may open up a whole new way of thinking about obesity research. The combination of tea and traditional Chinese medicine has a long history. The current version of the Chinese Pharmacopoeia contains a proprietary Chinese medicine, “Wu Shi Cha Granules” (Chinese pharmacopoeia commission, 2020a), which contains black tea in its formula. In contrast, another proprietary Chinese medicine, “Chuan Xiong Tea Tune Granules” (Chinese pharmacopoeia commission, 2020b), has a description in its usage and dosage section that states, “Take with strong tea after meals, one bag once, two times a day, reduce as appropriate for children.” The WLTT was conceived with this research in mind.

The research team conducted a systematic study on WLTT based on a high‐calorie obese rat model. The results showed that WLTT significantly reduced body weight, weight gain, Lee's index, visceral fat content, fat coefficient, and lipid TG levels in obese rats after 7 weeks of intervention compared to the HFD group. HE staining of adipose tissue also showed a significant decrease in adipocyte volume and number; antioxidant enzymes SOD and GSH‐Px were significantly increased, while MDA was significantly decreased. There was no significant reduction in the amount of food consumed by the rats in all intervention dose groups, and the results of the above experiments compared favorably with those of the BSY group in parallel experiments. All these studies show that WLTT has an excellent preventive effect on obesity. Food safety is a constant focus worldwide (He & Shi, 2021; Rodríguez‐Pérez et al., 2019), and the safety of WLTT based on a new research idea is also a focus of the team. Analysis of liver indices, ALT, AST, and HE staining of liver tissues at the end of the experiment showed that WLTT could improve the liver function of rats in a hypercaloric model, with a potential protective effect. The absence of significant changes in kidney index and the downregulation of BUN and Cre indicators indicate that WLTT does not impair renal function. All these data indicate that WLTT has a good safety profile.

In recent years, intestinal flora has gained much attention as an essential target for chronic metabolic diseases (Cao et al., 2019; Du et al., 2021; Ma et al., 2019). Under normal conditions, the intestinal flora, consisting of trillions of bacteria, maintains a symbiotic relationship with its host and plays a role in the metabolic and energy regulation of the body. Numerous studies have demonstrated that the alteration of the intestinal flora in chronic metabolic diseases mainly increases the abundance of the *Firmicutes* and decreases the abundance of the *Bacteroidetes*. The ratio of the *Firmicutes* to the *Bacteroidetes* (*Firmicutes/Bacteroidetes, F/B*) has become one of the leading reference indicators for studying intestinal flora disorders (Indiani et al., 2018; Xue et al., 2016). The 16S rRNA high‐throughput sequencing showed that WLTT improved the structure of the intestinal flora of the high‐calorie model rats, downregulating the abundance of *Firmicutes* and slightly increasing the abundance of *Bacteroidetes* while upregulating the relative abundance of *Verrucomicrobia*. However, the group was puzzled by the fact that none of the experimental groups showed high‐abundance values of Bacteroidetes in this experiment, which could be related to the source of the experimental intestinal bacteria samples to the cecum contents.

Probiotics have been shown to combat the development of some chronic metabolic disorders because of their beneficial regulation of the intestinal flora (Ji & Shen, 2021; Mohamad Nor et al., 2021; Mularczyk et al., 2021; Rittiphairoj et al., 2021; Tillmann et al., 2021). *Akkermansia muciniphila*, a normal flora of the human intestine, is the best‐known probiotic in *Akkermansia*, and numerous studies have shown a negative association between *Akkermansia muciniphila* and diseases such as obesity, diabetes, cardiovascular disease, and low‐grade inflammation (Higarza et al., 2021; Zhang et al., 2021). *Lachnospiraceae* has also been shown to reduce obesity and improve inflammation as a potentially beneficial bacterium (Naudhani et al., 2021; Truax et al., 2018). The group found that WLTT and BSY significantly increased the relative abundance of *Akkermansia, unclassified_Lachnospiraceae* at the genus level, suggesting that WLTT and BSY can positively regulate probiotic disorders in the intestinal flora caused by a high‐calorie diet. At the same time, studies have confirmed that *Romboutsia* and *Ruminococcus* are positively correlated with waist circumference and body mass index, LDL, TG, and TC indicators of blood lipids (Wang, Ablimit, et al., 2021; Yuan et al., 2021; Zeng et al., 2019) The group results showed that the relative abundance of *Romboutsia* and *Ruminococcus* was also significantly downregulated by the intervention of WLTT and BSY. In addition, the relative abundance of *Saccharibacteria_genera_incertae_sedis* and *Corynebacterium* flora also showed a significant decreasing trend. It can be seen that both WLTT and BSY have an excellent ability to regulate the disturbance of intestinal flora caused by a high‐calorie diet. However, after an in‐depth comparison of the ability of WLTT and BSY to intervene in the intestinal flora, it was found that *Psychrobacter* and *Aerococcus* showed a significant upward trend in the BSY intervention group. In contrast, no statistical difference was shown between the experimental groups of WLTT, while *Aerococcus* was confirmed as a human pathogen in numerous studies (Rasmussen, 2016; Tai et al., 2021), which led the group to conclude that there is some risk potential in the regulation of flora by BSY.

The group compared the species composition obtained from sequencing with the KEGG PATHWAY database and found that the intervention of the WLTT groups led to changes in the functional abundance of microorganisms, and the number of statistically different functional metabolic pathways increased with increasing dose. It mainly causes changes in some metabolic pathways, such as amino acid, insulin, and bile metabolism, in addition to altering the biosynthesis and degradation of some secondary metabolites, which are mostly positively associated with obesity and lipid metabolism.

## 5 CONCLUSIONS

In summary, WLTT has a significant preventive effect on a high‐calorie model of obesity in rats, while the drink also has a good safety profile. The regulation of the intestinal flora significantly, the increase in the abundance of *Akkermansia* and *unclassified_Lachnospiraceae*, and the decreases in the abundance of *Romboutsia* and *Ruminococcuse*, thus affecting changes in metabolic pathways such as amino acid, insulin, and bile metabolism and the biosynthesis and degradation of secondary metabolites, may be the key reasons for the effectiveness of WLTT. The combination of Tibetan Tea and medicine food homology herbs may be superior to green tea and medicine food homology herbs in terms of function and safety, which could be a new strategy for obesity prevention.

## CONFLICT OF INTEREST

The authors declare no conflicts of interest.
